# Effect of Lysosomal Cathepsin L on Proteolysis of Beef Myofibrillar Proteins In Vivo and In Vitro

**DOI:** 10.3390/foods11040613

**Published:** 2022-02-21

**Authors:** Baowei Cui, Xiuyun Guo, Yawei Zhang, Xiangren Meng

**Affiliations:** 1 Faculty of Food Science and Technology, Suzhou Polytechnic Institute of Agriculture, Suzhou 215008, China; cuibaow110@126.com; 2 School of Tourism and Cuisine, Yangzhou University, Yangzhou 225127, China; xrmeng@yzu.edu.cn; 3 Key Laboratory of Chinese Cuisine intangible Cultural Heritage Technology Inheritance, Ministry of Culture and Tourism, Yangzhou 225127, China; 4 National Center of Meat Quality and Safety Control, College of Food Science and Technology, Nanjing Agricultural University, Nanjing 210095, China; zhangyawei@njau.edu.cn

**Keywords:** cathepsin L, proteolysis, tenderness, beef, western blotting

## Abstract

This study investigated the effects of cathepsin L on proteolysis of beef myofibrillar proteins in vivo and in vitro. Results indicated that cathepsin L affected the degradation of desmin and troponin-T during postmortem aging, and the extent of degradation increased from 1 d to 14 d postmortem. No detectable degradation of titin, nebulin, and α-actinin in the presence of cathepsin L inhibitor was observed during postmortem aging. In vitro, cathepsin L affected the degradation of titin, nebulin, and troponin-T, and the extent of degradation increased with increasing incubation time. Nevertheless, cathepsin L did not cause the degradation of α-actinin and desmin, regardless of incubation temperature. The different results between in vitro and in vivo experiments might mainly depend on different treatment temperatures. Overall, these results indicated that cathepsin L participated in the degradation of myofibrillar proteins and meat tenderization.

## 1. Introduction

Tenderness is generally considered to be the most important sensorial attribute for consumers regarding meat consumption [1,2]. Therefore, improving the tenderness of meat is an important issue in the meat industry [3]. Meat tenderness is mainly determined by the proteolysis of key myofibrillar and cytoskeletal proteins, such as titin, nebulin, α-actinin, desmin, troponin-T, dystrophin, and vinculin during the postmortem aging [4,5,6]. Endogenous proteolytic enzymes (e.g., calpains, caspases, and lysosomal proteinases) are of crucial importance for the postmortem proteolysis of the key myofibrillar and cytoskeletal proteins, and thus, in meat tenderization [7,8]. Cathepsins, which are located in the lysosomes of muscle cells, and potentially released during postmortem aging, are favored by postmortem cells, and play an important role in proteolysis and meat tenderization [9]. 

However, the effect of cathepsins on meat tenderness varies considerably among studies. Previous studies have shown that cathepsin L (CAT) can degrade myofibrillar proteins (MPs) in vitro [8,10]. Myosin heavy chain (MHC), α-actinin, actin, troponin-T, and troponin-I assembled in rabbit myofibrils are degraded by CAT at 37 °C according to the study by Matsukura et al. [11]. Mikami et al. [12] also find that CAT hydrolyzed most myofibrillar proteins, including titin, nebulin, troponin-T, and tropomyosin in beef, rabbit, and chicken myofibrils in vitro. Nevertheless, several studies have shown that CAT has no effect on MPs proteolysis and meat tenderness in vivo. For instance, the results of Uytterhaegen et al. [9] indicate that CAT plays no significant role in MPs degradation at 2 °C during 8 d postmortem. In addition, Koohmaraie et al. [13] also conclude, from sodium dodecyl sulphate-polyacrylamide gel electrophoresis (SDS-PAGE) patterns, that CAT has no contribution to the degradation of MPs. The different treatment temperatures seem to be responsible for the contradictory results of MPs degradation by CAT in vivo and in vitro. Nevertheless, this hypothesis has yet to be verified. Therefore, to understand the role of CAT in meat tenderness, it is necessary to explore the effect of CAT on proteolysis both in vivo and in vitro. 

The aim of the present study was to investigate the effect of CAT on the proteolysis of MPs both in vivo and in vitro. 

## 2. Materials and Methods

Purified CAT from a bovine pancreas was used to examine the effect of CAT on beef myofibrils at different temperatures in vitro, and selective CAT inhibitor was chosen to investigate the degradation of CAT on bovine skeletal muscle protein during postmortem aging.

### 2.1. In Vivo Experiments

#### 2.1.1. Sample Preparation

Three 2.5 years old Simmental Crossbred cattle (live weight 428 ± 38 kg) were selected from a farm at Hebei Fucheng Food Co., Ltd (Yanjiao, China). After 24 h of rest, they were humanely slaughtered on the basis of the Operating Procedures of Cattle Slaughter in the National Standards of China. At approximately 30–45 min after exsanguination, the *longissimus thoracis* muscles (from 5th lumbar vertebrae to 12th thoracic vertebrae) were removed from the carcass, and excess fat was trimmed. About 100 g of muscle was collected as 0 day samples, and immediately frozen in liquid nitrogen, and stored until subsequent analysis. Approximately 120 g of muscle was cut into small pieces, and divided into three groups (ca. 40 g/group). Each group of muscle was then subdivided into four fractions (ca. 10 g/fraction), and soaked in the following treatment buffer, in the ratio of 1:1 (*w*/*v*) (meat/solution): (1) control: 60 mM NaCl and 2 mM NaN_3_ (C); (2) control + 100 μM cathepsin L inhibitor (CATI, Sigma-Aldrich, Milwaukee, WI, USA), and then stored for 1, 3, 7, and 14 day at 4 °C. Afterwards, samples were collected individually, and snap-frozen in liquid nitrogen, and then stored at −80 °C until required.

#### 2.1.2. Extraction of MPs

MPs were extracted according to the method described by Huang et al. [14], with some modifications. Samples were processed at 0–4 °C to minimize the proteolysis or protein denaturation. Briefly, about 1.0 g of minced muscle was homogenized in 8 mL of PRB buffer (100 mM KC1, 2 mM MgCl_2_, 2 mM EDTA, 1 mM DTT, 1 mM NaN_3_, 2 mM Na_4_P_2_0_7_, and 10 mM Tris-maleate, pH 6.8, 4 °C) using a polytron at a speed of 14,000 rpm for 30 s, with an interval of 15 s between bursts. After being centrifugated at 1000× *g* for 10 min, the supernatant was decanted, and the pellet was resuspended in 10 mL of low salt buffer (100 mM KC1, 2 mM MgCl_2_, 2 mM EDTA, 1 mM DTT, 1 mM NaN_3_, and 10 mM Tris-maleate, pH 6.8, 4 °C). Then, the centrifugation and resuspension processes were repeated six times. Finally, the pellet was washed twice with 10 mL Tris-EDTA buffer (10 mM Tris-HCl and 5 mM EDTA, pH 8.0, 4 °C). The protein concentration was determined using the BCA Protein Assay Kit. 

MPs extracted from different samples were immediately diluted to 6 mg/mL, and then mixed with buffer (30 mM Tris-HCl, 3 mM EDTA, 3% SDS, 20% glycerol, 8% 2-mercaptoethanol, and 0.04% Bromophenol blue, pH 8.0) at a ratio of 1:1 (*v*/*v*). Next, the samples were heated in a 50 °C water bath for 20 min, and then centrifuged at 10,000× *g* for 20 min at 4 °C. Finally, samples were stored at −80 °C for SDS-PAGE and western blotting.

### 2.2. In Vitro Experiments

#### 2.2.1. Preparation of CAT

CAT was purified from a bovine pancreas according to the procedure reported by Li et al. [15], with little modification. Fat and connective tissue were removed from a fresh bovine pancreas at 4 °C. The minced bovine pancreas was homogenized with four volumes of extraction buffer (25 mM sodium acetate buffer, 5 mM L-Cys and 0.3 mM PMSF, pH 5.0) for 2 min. The samples were then centrifuged at 10,000× *g* for 20 min to obtain a crude enzyme solution, whose pH was adjusted to 3.0 using 1 M HCl. Next, the solution was incubated at 30 °C for 10 min, and then centrifuged at 12,000× *g* for 20 min immediately after adjusting the pH to 5.8–6.0 using 1 M NaOH. The supernatant was obtained and then salted out with 80% ammonium sulfate, and centrifuged at 10,000× *g* for 20 min. Next, the sediment was collected and dialyzed against the phosphate buffer. The dialysis sample was concentrated using an Amicon Ultra-15 tube, and then passed through DEAE Sephacel, Sephacryl S-100, SP-Spharose FF, and Con A-Sepharose affinity chromatography columns. During purification, the hydrolytic activity of Z-Phe-Arg-MCA fluorescent substrate was monitored, and the active peaks were collected, concentrated, and stored at −80 °C. CAT activity was determined using the method of Wang et al. [16], with Z-Phe-Arg-MCA as the substrate. One unit of enzyme activity was defined as the amount of activity that released 1 nmol of AMC per min at pH 5.8 and 37 °C. 

#### 2.2.2. Incubation of MPs with Purified CAT

MPs extracted from 0 day samples were used to investigate the role of CAT in the degradation of MPs in vitro. MPs extracted from 0 d samples were set as the 0 h sample, and were diluted to a final concentration of 2.0 mg/mL. Then, fractions of MPs were incubated with CAT (25 U/mg of MPs) in incubation buffer (10 mM Tris-HCl and 5 mM EDTA, pH 5.8) at 4, 20, and 37 °C for 10 h, respectively). MPs incubated without CAT were set as control (C). After 10 h, the incubation was stopped by the addition of an equal volume of sampling treatment buffer (30 mM Tris-HCl, 3 mM EDTA, 3% SDS, 20% glycerol, 8% 2-mercaptoethanol, and 0.04% bromophenol blue, pH 8.0). Samples were immediately denatured at 100 °C for 5 min, and then stored at −80 °C for SDS-PAGE and western blotting.

### 2.3. SDS-PAGE and Western Blotting

Proteins were resolved with SDS-PAGE, and western blotting was conducted as described by Carlson et al. [17], with some modification. A 5% polyacrylamide slab separating gel, without a stacking gel, was used to examine the changes in titin and nebulin integrity. For 5% gels, 70 μg of MPs samples were loaded per lane, and run at a constant current of 4.5 mA per gel for 15 h using the Bio-Rad Min-Protean II system (Bio-Rad Laboratories, Hercules, CA, USA). Conversely, 10% and 12.5% polyacrylamide separating gels with a 5% polyacrylamide stacking gel were used to monitor the changes in α-actinin, desmin, and troponin-T. MPs samples (40 μg) were loaded per well to detect α-actinin and desmin, whereas 20 μg were used for troponin-T. All samples were run at a constant voltage of 80 V for stacking gels, and 100 V for separating gels at room temperature (25 °C). Triplicate gels were run, and the running buffer contained 25 mM Tris, 192 mM glycine, and 0.1% SDS. After electrophoresis, gels were either stained for the examination of all protein bands, or transferred by polyvinylidene difluoride (PVDF, Bio-Rad Laboratories, Hercules, CA, USA) membranes for western blotting. Gels were stained for 6 h with 0.1% Coomassie Brilliant Blue R-250 (*w*/*v*), 40% ethanol (*v*/*v*), and 7% glacial acetic acid (*v*/*v*). After staining, the same solution without Coomassie Brilliant Blue R-250 was used to destain the gels. Gels for western blotting were immediately transferred to PVDF membranes using the Bio-Rad Min-Protean II system at 90 V for 2 h for α-actinin, 80 V for 1 h for desmin, and 70 V for 1 h for troponin-T, respectively. The transferring buffer contained 25 mM Tris, 192 mM glycine, and 10% methanol (*v*/*v*). The electro-blotted membranes were then incubated at 4 °C for 12 h in TTBS blocking buffer (0.05% TWEEN 20 (*w*/*v*), 20 mM Tris, 137 mM NaCl, 5 mM KCl, and 5% skim milk (*w*/*v*)). After blocking, the membranes were then incubated with mouseanti-α-acitinin monoclonal antibody (Abcam, Cambridge, UK) at a dilution of 1:250, mouse anti-desmin monoclonal antibody diluted 1:400 (Sigma-Aldrich, Milwaukee, WI, USA), and mouse anti-troponin-T monoclonal antibody at 1:500 (Sigma-Aldrich, Milwaukee, WI, USA), respectively. After three washes with TTBS buffer for 10 min, the membranes were incubated with goat anti-mouse IgG horseradish peroxidase, conjugated affinity purified secondary antibody at a dilution of 1:5000 for α-actinin (Abcam, Cambridge, UK), 1:5000 for desmin (Sigma-Aldrich, Milwaukee, WI, USA), and 1:10,000 for troponin-T (Sigma-Aldrich, Milwaukee, WI, USA), respectively. Membranes were rinsed thrice in TTBS buffer for 10 min before detection. A chemiluminescent detection system (GT-800F EPSON) was used to detect the immunoreactive protein bands, and the densities of targeted bands were analyzed by Quantity One software (Bio-Rad Laboratories).

### 2.4. Statistical Analysis

The experiments were performed in triplicate. The data were analyzed using a Statistical Analysis System (SAS Institute Inc., Cary, NC, USA). One-way ANOVA with Duncan’s multiple range test was performed to measure the significant differences between samples (*p* < 0.05). 

## 3. Result and Discussion 

The tenderization of meat is a complex interaction of biochemical processes, among which, protein degradation by endogenous enzymes plays a pivotal role [5,6]. The proteolysis of key skeletal and costamere MPs, such as titin, nebulin, α-actinin, desmin, and troponin-T, has been reported to be related to the tenderness of aged meat [18].

### 3.1. Titin and Nebulin

**Figure 1:**
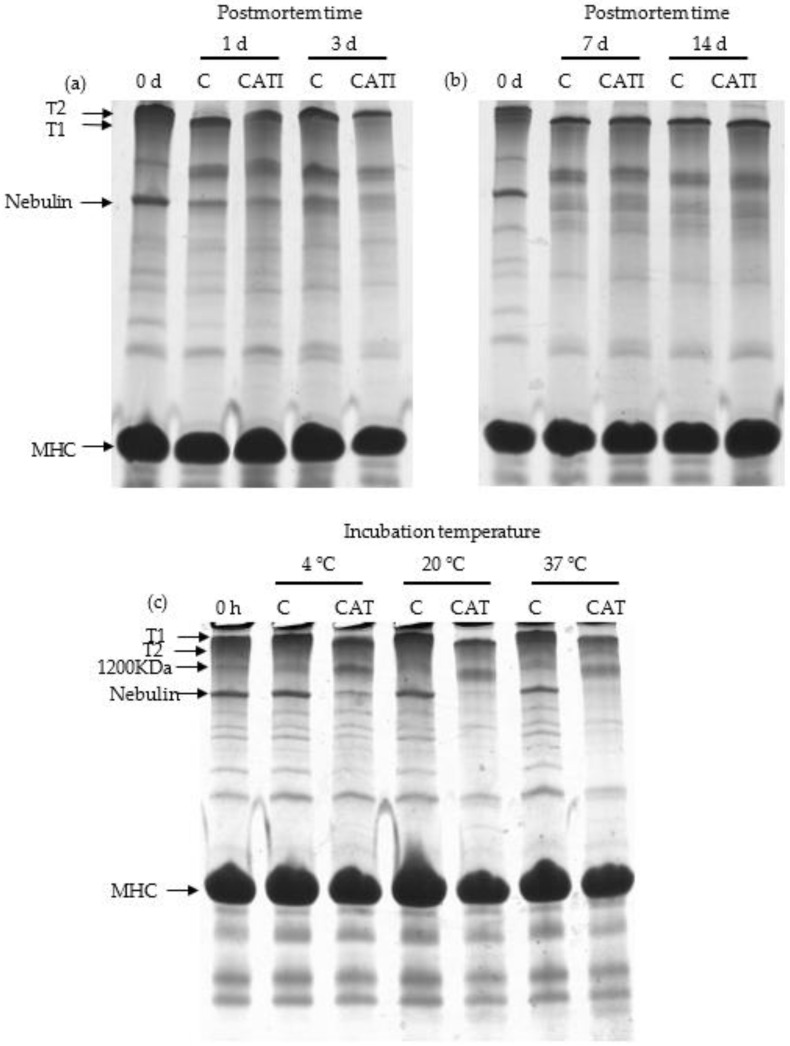
SDS-PAGE patterns of Coomassie-stained 5% continuous polyacrylamide gel, indicating the degradation of titin and nebulin by cathepsin L in vivo and in vitro. (**a**) Samples treated with cathepsin L inhibitor for 0, 1, and 3 day postmortem at 4 °C in vivo; (**b**) samples treated with cathepsin L inhibitor for 0, 7, and 14 day postmortem at 4 °C in vivo; (**c**) samples incubated with cathepsin L for 10 h at 4, 20, and 37 °C in vitro. Abbreviations are as follows: T1 = intact titin; T2 = degradation product of intact titin; MHC = myosin heavy chain; C = control; CAT = cathepsin L; CATI = sample incubated with cathepsin L inhibitor.

Titin (approximately 3000 kDa) and nebulin (approximately 800 kDa), anchoring one of their ends to the Z-line, are considered to be important contributors to myofibril integrity and meat tenderness [19,20,21]. The effect of CAT on hydrolysis of titin and nebulin in vivo and in vitro is shown in Figure 1. For the 0 day samples, a major band for intact titin (T1) was observed. After 1 day postmortem, a major band for T1 degradation was observed, marked as T2 (approximately 2400 kDa), which was in accordance with the results reported by Taylor et al. [18]. Nevertheless, there was no observable difference between the various postmortem aging times. Regarding the T2 bands at 1, 3, 7, and 14 day postmortem, no obvious difference (*p* > 0.05) was found between the control and samples treated with CATI. In contrast to that of titin, the intensity of the nebulin band decreased (*p* < 0.05) as postmortem aging time increased. In particular, the intact nebulin band had nearly disappeared after 7 day postmortem. However, no obvious changes were found between the control and samples treated with CATI during the postmortem. These results indicated that CAT had no effect on the degradation of titin and nebulin during postmortem aging. 

Figure 1c shows the results of purified MPs incubated with CAT at 4, 20, and 37 °C for 10 h. There were no notable changes (*p* > 0.05) in the band for both titin and nebulin between unincubated MPs and control samples. In addition, T1 was degraded to T2 after being incubated with CAT, and the intensity of the band for T1 was gradually decreased (*p* < 0.05) with increasing temperature. At 37 °C, T1 was totally degraded by CAT. Similar to titin, obvious differences (*p* < 0.05) in nebulin were observed between the control and samples incubated with CAT. Besides, it could be also found that the extent of nebulin degradation was increased (*p* < 0.05) with increasing temperature. The different results between in vivo and in vitro experiments might be due to the different temperatures. It is known that enzyme activity increases with increasing temperature, and as such, the lower temperature might be one factor resulting in the resistance to the degradation of titin and nebulin in vivo.

### 3.2. α-Actinin

**Figure 2:**
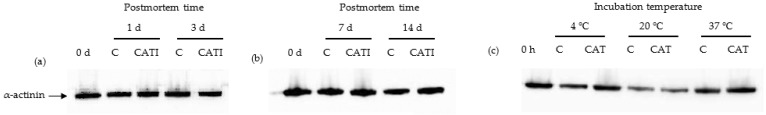
Representative western blotting patterns showing the degradation of α-actinin by cathepsin L in vivo and in vitro. (**a**) Samples treated with cathepsin L inhibitor for 0, 1, and 3 day postmortem at 4 °C in vivo; (**b**) samples treated with cathepsin L inhibitor for 0, 7, and 14 day postmortem at 4 °C in vivo; (**c**) samples incubated with cathepsin L for 10 h at 4, 20, and 37 °C in vitro.

α-Actinin is crucial for connecting together actin filaments from adjacent sarcomeres, and forming the Z-disk, and then contributing to proper muscle physiology [22]. Changes of α-actinin caused by CAT in vitro and in vivo were investigated (Figure 2). As shown in Figure 2a,b, proteolytic fragments of α-actinin were not detected in all bands when using anti-α-actinin monoclonal antibody in vivo. This was in accordance with the study of Ho et al., who revealed that little α-actinin change was detected using western blot analysis through 28 d of bovine longissimus muscle postmortem aging [23]. Also, the degradation fraction of α-actinin was not detected in all samples in vitro (Figure 2c). Taken together, CAT had no effect on the degradation of α-actinin both in vivo and in vitro. 

### 3.3. Desmin

**Figure 3:**
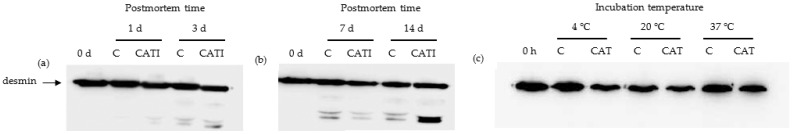
Representative western blotting patterns showing the degradation of desmin by cathepsin L in vivo and in vitro. (**a**) Samples treated with cathepsin L inhibitor for 0, 1, and 3 day postmortem at 4 °C in vivo; (**b**) samples treated with cathepsin L inhibitor for 0, 7, and 14 day postmortem at 4 °C in vivo; (**c**) samples incubated with cathepsin L for 10 h at 4, 20, and 37 °C in vitro.

Desmin comprises mostly attachments of Z- to Z-line, and is likely a key substrate that determines meat tenderness [19]. The effect of CAT on the degradation of desmin in vivo is shown in Figure 3a,b. In contrast to α-actinin, compared with the 0 day sample, the degradation fragment of desmin was observed during postmortem aging. For the control sample, one new faint band and two new faint bands were observed at 1 day postmortem and after 3 day postmortem, respectively. This was in agreement with the study of Hwan, which indicated that desmin was easily degraded at 4 °C in bovine semitendinosus muscle during the aging process [24]. In addition, the intensity of the new band was increased (*p* < 0.05) with an increased postmortem time. Similarly, two new faint bands were observed, whose intensity was increased (*p* < 0.05) with increased postmortem time for samples treated with CATI. Moreover, it was obvious that the intensity of the new band for samples treated with CATI was higher (*p* < 0.05) than that of the control. These results indicated that CAT participated in the degradation of desmin during postmortem aging. 

The effect of CAT on the degradation of desmin in vitro is shown in Figure 3c. Compared with the 0 day sample, the degradation fragment of desmin was not detected in the control sample at 4, 20, and 37 °C, indicating that temperature did not affect the degradation of desmin. In addition, compared with the control sample, the degradation fragment of desmin was also not detected in the CAT-treated samples, regardless of incubation temperature. These results indicated that CAT did not participate in the degradation of desmin in vitro.

### 3.4. Troponin-T

**Figure 4:**
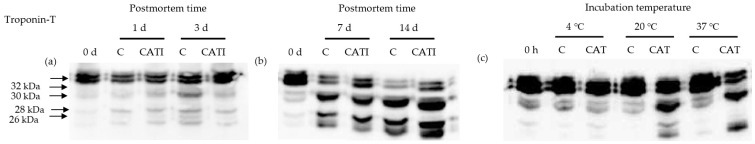
Representative western blotting patterns showing the degradation of troponin-T by cathepsin L in vivo and in vitro. (**a**) Samples treated with cathepsin L inhibitor for 0, 1, 3 day postmortem at 4 °C in vivo; (**b**) samples treated with cathepsin L inhibitor for 0, 7, 14 day postmortem at 4 °C in vivo; (**c**) samples incubated with cathepsin L for 10 h at 4, 20, and 37 °C in vitro.

Troponin-T is the tropomyosin-binding component of the troponin complex, and is generally considered to be the most proteolytically sensitive subunit of the troponin molecule [25]. Degradation of troponin-T is considered to be an indicator of meat aging and tenderization [26,27], as denoted by the appearance of the main proteolysis fragments of 28–32 kDa [12,27,28,29]. Representative western blotting patterns are shown in Figure 4a,b, and the lanes indicate that the anti-troponin-T monoclonal antibody could clearly and strongly recognize the troponin-T bands. At 0 day postmortem, the 28, 30, and 32 kDa bands were detected, which might be mainly due to the part degradation of troponin-T in the extracting process of samples. Compared with the 0 day sample, a new 26 kDa band was detected during postmortem aging from 1 day to 14 day. In addition, the intensity of 26–32 kDa bands was gradually increased (*p* < 0.05) with increased postmortem time for both control and CATI-treated samples, accompanied by a decrease in the intensity of troponin-T band. In addition, there was no obvious change (*p* > 0.05) between the control and CATI-treated samples at 1 day and 3 day postmortem. Nevertheless, compared with the control, the degradation of troponin-T treated with CATI was more obvious after 7 day postmortem, and the intensity of 26–32 kDa bands was higher than that of the control samples. These results indicated that CAT participated in the degradation of troponin-T during postmortem aging. 

The effect of CAT on the degradation of troponin-T in vitro is shown in Figure 4c. At 4 °C, there was no observable difference (*p* > 0.05) between control and CAT-treated samples. At 20 °C, the 28, 30, and 32 kDa bands were detected in the control sample, whereas a new 26 kDa band was detected in the CAT-treated sample. Furthermore, the intensity of degraded fraction bands in the CAT-treated sample was higher (*p* < 0.05) than that in the control sample. At 37 °C, only 26, 28, and 30 kDa bands were detected in the CAT-treated sample. Similarly, the intensity of bands in the CAT-treated sample was higher (*p* < 0.05) than that in the control sample. These results indicated that CAT affected the degradation of troponin-T in vitro, and the extent of the degradation was affected by incubation temperature. This was in accordance with the results of previous studies showing that cathepsin L could hydrolyze troponin-T from rabbit or carp myofibril protein at 20 and 37 °C [11,30]. In summary, CAT participated in the degradation of troponin-T both in vitro and in vivo.

Based on the results of the degradation of titin, nebulin, α-actinin, desmin, and tropinin-T in in vitro and in vivo experiments, we could conclude that CAT had an important role in degrading MPs in vivo and in vitro, and thus, might contribute to meat tenderization, which was confirmed by the results of the shear force test, as shown in the Supplementary Materials.

## 4. Conclusions

This study demonstrated that CAT participated in the degradation of MPs both in vivo and in vitro. CAT caused the degradation of titin and nebulin in vitro, the extent of which increased with increased incubation temperature, but had no effect on the degradation of these MPs in vivo. In addition, compared with the control, the presence of CATI caused further degradation of desmin in vivo, the extent of which was increased with increased postmortem aging time, whereas no degradation of desmin was observed in the presence of CAT in vitro, regardless of incubation time. For troponin-T, compared with the control, CATI did not cause an obvious degradation of troponin-T at 1 and 3 d postmortem, but exerted more obvious degradation after 7 day postmortem in vivo. In addition, the presence of CAT caused further degradation of troponin-T in vitro, and the extent of the degradation was increased with increased incubation temperature. Temperature might be a critical factor causing the difference between in vivo and in vitro results. Therefore, the results might provide a reference for understanding the role of CAT in MPs degradation and meat tenderization.

## Data Availability

The data that support the findings of this study are available on request from the corresponding author.

## Supplementary Materials

The following supporting information can be downloaded at: https://www.mdpi.com/article/10.3390/foods11040613/s1, Figure S1: Warner-Bratzler shear force of beef treated with cathepsin L in different incubated temperature.
