# Supplementary material for: Effect of Lysosomal Cathepsin L on Proteolysis of Beef Myofibrillar Proteins In Vivo and In Vitro
Source: Foods. 2022 Feb 21;11(4):613. doi: 10.3390/foods11040613 (PMC8871152; doi:10.3390/foods11040613)
Supplement: Supplementary file 1 [file foods-11-00613-s001.zip › foods-1579602-supplementary.pdf]

1. Material and Method

1.1 Preparation of samples

longissimus thoracis muscles were incubated with CAT (100 U/g *longissimus thoracis* muscles) in incubation buffer (10 mM Tris-HCl, 5 mM EDTA, pH 5.8) at 4 °C, 20 °C and 37°C for 10 h, respectively. Muscles incubated without CAT were set as control.

1.2 Measurement of Warner-Bratzler shear force (WBSF)

The measurement of WBSF was determined. In brief, samples were placed in plastic bags and immersed completely in a 70 °C water bath until the core temperature reached 70 °C. After cooking, samples were cooled to room temperature. Six cores with the diameter of 1.27 cm were removed parallel to the muscle fiber orientation from each sample and were measured by a texture analysis machine (TA-XT2i, Stable Miroc System, England) equipped with a HDP/BSW blade. The parameters were set as: pre-test speed of 2.0 mm/s, test speed of 1.0 mm/s, post-test speed of 5.0 mm/s, and the distance is 23.0 mm, trigger type is auto-40 g. The average measurement of cores from each steak was the value of WBSF expressed in Newtons (N).

2. Result and Discussion

The effect of CAT on the WBSF of samples incubated with different temperatures was shown in Fig. 5S. It was shown that there was no significant change ($p > 0.05$) among control samples treated with different temperature, indicating that temperature didn't have a direct effect on the WBSF of samples. In contrast, the WBSF of samples treated with CAT was decreased ($p < 0.05$) with increasing incubated temperature. At 37 °C, compared with control, the WBSF of samples treated with CAT was decreased ($p < 0.05$) by 35.59%. These results indicated that the presence of CAT affect the WBSF of beef muscle depending on the incubated temperature. As known, the activity of CAT was increased with increasing temperature, facilitating the hydrolysis of MPs, thus decreasing the WBSF and improving meat tenderness.

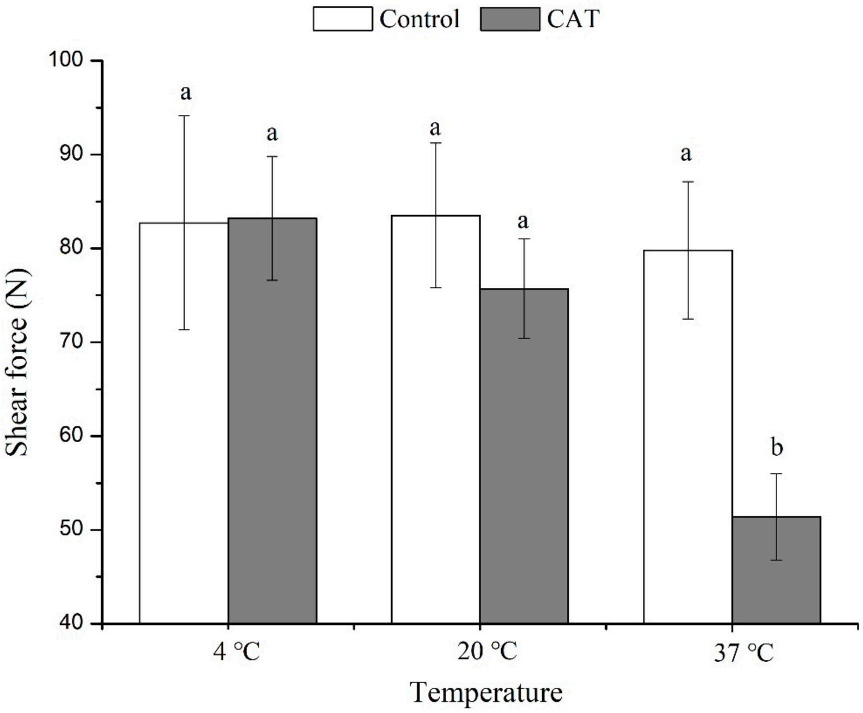

Figure S1: Warner-Bratzler shear force of beef treated with cathepsin L in different incubated temperature
